# Conformational stability and domain‐specific structural features of tumor autoantigens regulate autoantibody epitope propensity

**DOI:** 10.1002/pro.70808

**Published:** 2026-09-23

**Authors:** Ai Miyamoto, Rika Yamamoto, Ryui Sakaguchi, Rikako Kutsuma, Takeru Mori, Mirei Masui, Tomoko Honjo, Midori Futami, Mariko Morii, Hiromi Watanabe, Tadahiro Kuribayashi, Kadoaki Ohashi, Katsuyuki Kiura, Junichiro Futami

**Affiliations:** ^1^ Graduate School of Interdisciplinary Science and Engineering in Health Systems Okayama University Okayama Japan; ^2^ Department of Bioscience, Faculty of Life Science Okayama University of Science Okayama Japan; ^3^ Department of Respiratory Medicine Okayama University Hospital Okayama Japan

**Keywords:** autoantibody, biomarker, cancer/testis antigen, epitope propensity, intrinsically disordered regions, protein engineering, tumor‐associated antigens

## Abstract

Autoantibodies against tumor‐associated autoantigens are clinically valuable biomarkers for cancer diagnosis; however, the structural determinants governing epitope selectivity remain unknown. Here, we investigated whether the intrinsic conformational stability of target autoantigens regulates the epitope propensity of tumor‐associated autoantibodies in non‐small cell lung cancer. Using a dual‐antigen Luminex bead‐based assay that presents each autoantigen in both native and *S*‐cationized denatured forms, we directly compared IgG autoantibody reactivity toward conformational and linear epitopes across 11 autoantigens. Intrinsically disordered aggregation‐prone autoantigens, including cancer/testis antigens, NY‐ESO‐1, and XAGE‐1b, predominantly elicit linear epitope‐directed responses, whereas thermodynamically stable soluble autoantigens generally drive conformational epitope recognition. Intriguingly, for autoantigens harboring both ordered and disordered segments, the immune response is strictly guided by domain‐specific biophysics: while p53‐specific antibodies preferentially target their flanking disordered regions, the Wilms' tumor protein 1 exceptionally drives conformational recognition directed toward its structured zinc‐finger domains. Recombinant solubility in *Escherichia coli* broadly correlated with epitope class across all 11 autoantigens, confirming that prokaryotic folding efficiency generally reflects intrinsic conformational stability in vivo for autonomously folding monomeric cytosolic proteins. Independent computational validation was provided by the concordance between the experimental solubility ratios and AlphaFold3‐derived Rosetta energy unit/solvent‐accessible surface area values, demonstrating the convergence of thermodynamic estimates and patient‐derived immune data within a unifying biophysical framework. These findings establish that the autoantibody epitope propensity is closely associated with the thermodynamic stability of the target autoantigen and provide a rational basis for tailoring antigen preparation strategies for autoantibody‐based cancer diagnosis.

## 1 INTRODUCTION

Antibodies are key effectors of the adaptive immune system that enable the precise recognition and neutralization of pathogenic antigens. Their functional efficacy primarily depends on their binding affinity and specificity, making the isolation of high‐affinity clones a central goal in therapeutic antibody development (Chi et al., 2024; Fibriansah et al., 2021; Mikolajek et al., 2022). Since high‐affinity antibodies typically target stable conformational epitopes that elicit robust immune responses, studies have traditionally focused on these interactions. However, when an antigen with an intrinsically unstable three‐dimensional structure elicits a humoral immune response, a fundamental question arises: which type of epitope, conformational or linear, dominates the expanded antibody repertoire in vivo? This study aimed to address this question by examining the human autoantibodies elicited during antitumor immune responses using a model system.

Under normal physiological conditions, immune tolerance to self‐antigens is critical for safeguarding against autoimmunity (Song et al., 2024). However, the homeostatic equilibrium is disrupted in both autoimmune diseases and cancers, leading to the generation of autoantibodies against self‐proteins (Ma et al., 2022). These autoantibodies are believed to arise from prolonged inflammation or antitumor immune responses, which promote the release of intracellular proteins and trigger humoral responses (Burbelo et al., 2021; Suurmond & Diamond, 2015). Since autoantibody production requires antigen stimulation of B cells (McHeyzer‐Williams et al., 2011) and the antigen is released from dead cells via antitumor immune responses or autoimmune reactions, the level of autoantibodies in the peripheral blood reflects the extent of these immune reactions. Notably, patients who respond well to immunotherapy show an autoantibody spark as a secondary immune response (Miyamoto et al., 2022; Mori et al., 2025). Although autoantibody targets vary among individuals, specific subsets have been established as clinically validated serum biomarkers (Sciascia et al., 2023; Wei et al., 2025). Systematic identification of autoantigens in patient serum is routinely performed using serological proteomics and protein microarray technologies (Beutgen et al., 2019; Carlton et al., 2023; Date et al., 2023; Kuzumi et al., 2023; Takeda, 2024; Tan et al., 2009).

Several molecular mechanisms have been proposed to explain the induction of autoantibody response. The prevailing hypothesis posits that certain autoantigens harbor virus‐like sequences that can activate autoreactive B cells from the naïve repertoire (Cusick et al., 2012; Shome et al., 2022). Additional studies have identified physicochemical “propensity rules” based on amino acid composition—encompassing hydrophilicity, basicity, aromaticity, and backbone flexibility—that may favor B‐cell epitope formation (Berzofsky, 1985; Shome et al., 2022). Nonetheless, epitope mapping studies have consistently revealed substantial inter‐individual heterogeneity, with autoantibodies frequently recognizing distinct linear epitopes across patients (Grüner et al., 2026; Huang et al., 2024; Kawabata et al., 2007).

Beyond sequence‐level considerations, it is important to recognize that many autoantigens are conformationally unstable and prone to aggregation (Ahmadi et al., 2021). Cancer/testis antigens (CTAs), which are aberrantly expressed in tumor cells, are particularly enriched in intrinsically disordered regions (IDRs) and frequently aggregate upon overexpression in human cell lines (Ahmadi et al., 2021; Almeida et al., 2009; Rajagopalan et al., 2011). Similarly, tumor‐associated antigens (TAAs), which are overexpressed or aberrantly modified in tumor cells, also exhibit a high aggregation propensity (Carl et al., 2005; Cheever et al., 2009). Autoantibodies against CTAs and TAAs are clinically useful biomarkers commonly detected in patients (Cheever et al., 2009; de Jonge et al., 2021; Ohue et al., 2019; Sakaeda et al., 2024; Sakai et al., 2021; Takashi et al., 2021). The aggregative and disordered nature of these proteins is believed to underlie their immunogenicity and capacity to elicit humoral responses (Lundahl et al., 2021; Rosenberg, 2006).

Considering their inherent structural instability, solubilization is critical for preparing recombinant autoantigens for immunoassays. The wheat germ cell‐free expression system is widely used because of its efficiency in producing soluble proteins suitable for autoantibody screening using microarrays (Fogeron et al., 2021; Takai et al., 2010). An alternative strategy involves fusion of target proteins with solubility‐enhancing tags (Arakawa & Akuta, 2025). We have previously developed a cysteine‐specific *S*‐cationization approach to solubilize inclusion body (IB)‐derived proteins for autoantibody detection (Futami et al., 2015; Miyamoto et al., 2022; Mori et al., 2025; Sakaguchi et al., 2026), yielding water‐soluble, denatured, and full‐length antigens with high chemical stability and purity. As CTAs and TAAs are largely disordered and structurally labile, we hypothesized that these autoantigens exist in non‐rigid partially unfolded states when released from tumor cells during antitumor immune responses. Under these conditions, B‐cell clones that recognize linear epitopes may preferentially expand during the ensuing humoral response. However, if an otherwise unstable or aggregation‐prone antigen contains locally well‐folded structural domains, these regional pockets might still preserve conformational epitopes in vivo. The multiplexed *S*‐cationized antigen‐immobilized Luminex beads (MUSCAT) assay, which we have developed previously, provides high sensitivity and quantitative accuracy for autoantibody detection (Futami et al., 2015; Miyamoto et al., 2022; Mori et al., 2025; Sakaguchi et al., 2026). This platform demonstrates high technical reliability, with an intra‐assay coefficient of variation (CV) of less than 10% and an inter‐assay CV of approximately 20%, thereby meeting the established clinical diagnostic criteria (Sakaguchi et al., 2026). Nevertheless, whether autoantibodies in patients with cancer preferentially recognize native‐like conformations, partially folded intermediates, or fully disordered antigen states remains unclear.

In this study, we addressed this question by directly comparing autoantibody reactivity toward five representative autoantigens frequently detected in patients with non‐small cell lung cancer (NSCLC), each presented in either a native‐like conformation or a chemically denatured *S*‐cationized state. This comparative framework may provide new insights into how antigenic structural disorders shape autoantibody epitope selection, and more broadly, into the mechanisms via which the humoral immune system recognizes tumor‐derived self‐proteins.

## 2 RESULTS

### 2.1 Selection of autoantibody biomarkers for epitope propensity analysis

**FIGURE 1:**
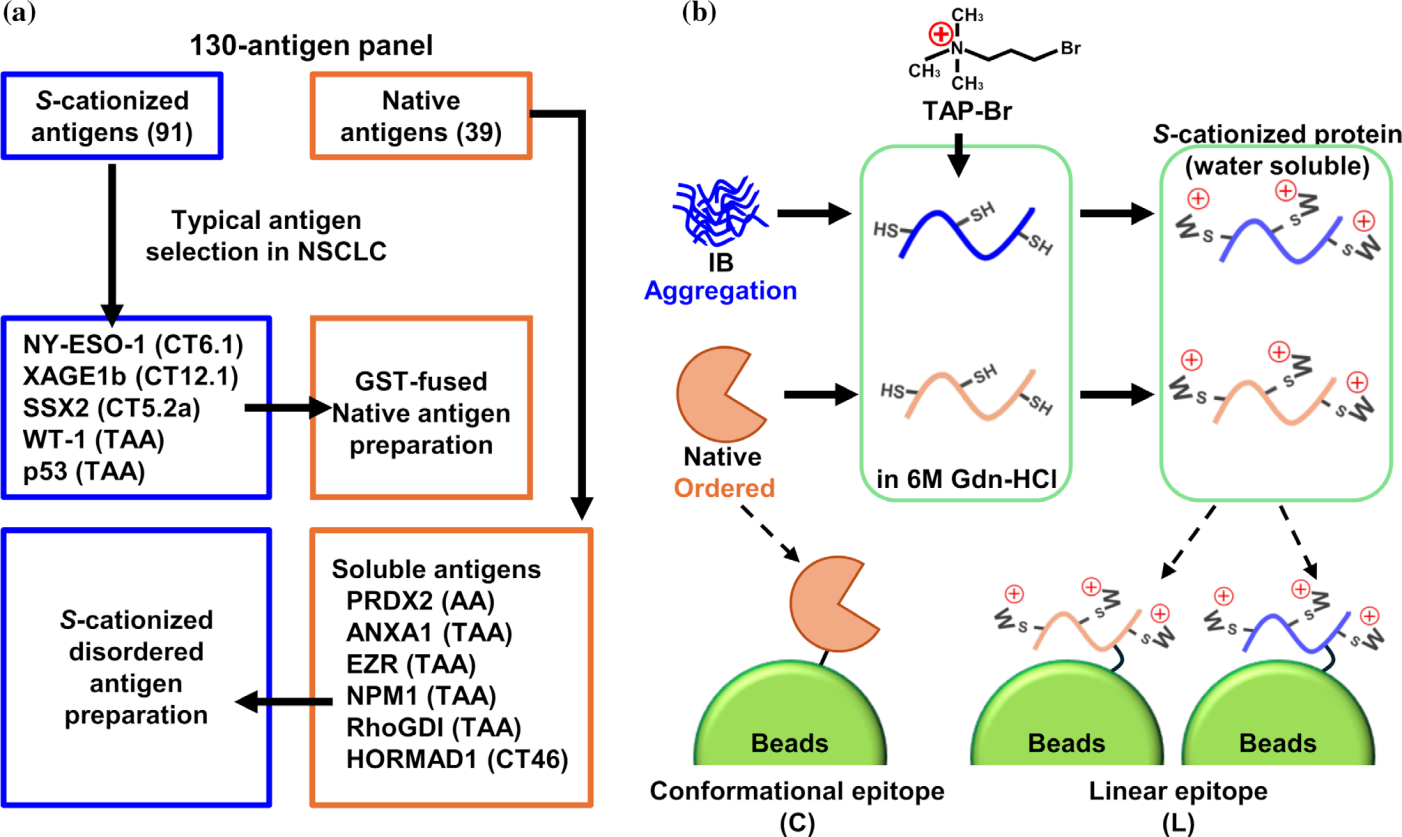
Conformational and linear epitope propensity analysis for autoantibody biomarkers in patients with non‐small cell lung cancer (NSCLC). (a) Autoantibody biomarkers were selected from the multiplexed *S*‐cationized antigen‐immobilized Luminex beads assay panel, which comprised 130 autoantigens. This panel includes 91 *S*‐cationized water‐soluble and denatured antigens derived from insoluble fractions and 39 native antigens purified from the soluble fraction of recombinant protein production systems. Five authentic autoantigens, commonly identified in NSCLC autoantibodies, were prepared as glutathione S‐transferase (GST)‐fused proteins to facilitate their expression in *Escherichia coli* as soluble proteins. Furthermore, six native antigens frequently observed in NSCLC autoantibodies were denatured, reduced, and *S*‐cationized to generate *S*‐cationized antigen counterparts for the assay. (b) Schemes for conformational (*C*) and linear (*L*) epitope analyses utilizing Luminex beads. GdnHCl, guanidinium hydrochloride; TAAs, tumor‐associated antigens; TAP‐Br, 3‐bromo‐*N*,*N*,*N*‐trimethylpropan‐1‐aminium bromide.

To investigate the linear and conformational epitope propensities, immunoglobulin G autoantibody biomarkers were selected from the profiles of patients with NSCLC and healthy donors using the MUSCAT assay panel, which analyzed 130 autoantigens (Mori et al., 2025). Elevated levels of five autoantibody biomarkers were identified in patients with NSCLC (Figure 1a and Table S1). These autoantibodies were selected based on their reactivity with *S*‐cationized denatured antigens immobilized on beads and were therefore classified as candidates with a preferential affinity for linear epitopes. The corresponding autoantigens were initially expressed as insoluble IBs in *Escherichia coli* (*E. coli*) and subsequently solubilized by *S*‐cationization via cysteine‐specific chemical modifications (Figure S1a). Cysteine‐specific *S*‐cationized proteins with 3‐bromo‐*N*,*N*,*N*‐trimethylpropan‐1‐aminium bromide (TAP‐Br) were designated as TAP‐modified antigens.

To obtain antigens in their native conformationally intact forms, they were expressed as glutathione S‐transferase (GST) fusion proteins under reduced‐temperature conditions (Figure S1a). Although all the constructs were successfully expressed as soluble proteins in *E. coli*, the autoantigen domains underwent various forms of proteolytic degradation owing to their instability. The resulting heterogeneity complicated purification on glutathione‐agarose columns; however, purification via streptactin‐affinity chromatography using a C‐terminal Strep‐Tag II fusion yielded full‐length proteins of sufficient purity. These soluble GST‐fused antigens were used to assess the conformational epitope‐binding properties of selected autoantibodies. Structural modeling based on the GST dimer interface (PDB ID: 6RWD) indicates that the C‐terminally fused autoantigen domains project outward in a trans orientation, minimizing steric hindrance and preserving autonomous domain folding and epitope accessibility.

The p53 autoantigen has a distinct structural organization, comprising intrinsically disordered N‐ and C‐terminal regions flanking a centrally located ordered DNA‐binding domain (DBD; residues 94–312) (Cho et al., 1994; Krois et al., 2018). As p53‐DBD was successfully expressed in soluble form in *E. coli*, this domain was also used as a probe for conformational epitope propensity analysis (Figure S1b).

Six autoantibody biomarkers that demonstrated preferential binding to native antigen conformations immobilized on the beads were identified as candidates with affinities for conformational epitopes (Figure 1a and Tabel S1). To enable a direct comparison of the relative binding propensities for conformational versus linear epitopes, native antigens were denatured using 6M guanidinium hydrochloride (GdnHCl), followed by *S*‐cationization, yielding *S*‐cationized denatured antigens (Futami et al., 2015; Miyamoto et al., 2022; Sakaguchi et al., 2026).

To directly compare the epitope propensities for autoantibody binding, both the conformationally native and *S*‐cationized linearized forms (Figure S1c) of each autoantigen were immobilized on spectrally distinct color‐coded Luminex beads (Futami et al., 2015). This dual‐antigen bead format enabled simultaneous assessment of autoantibody reactivity toward conformational and linear epitopes in a single multiplexed assay, facilitating direct structural comparisons across autoantigens (Figure 1b).

### 2.2 Comparative analysis of epitope propensity for IgG autoantibody binding

**FIGURE 2:**
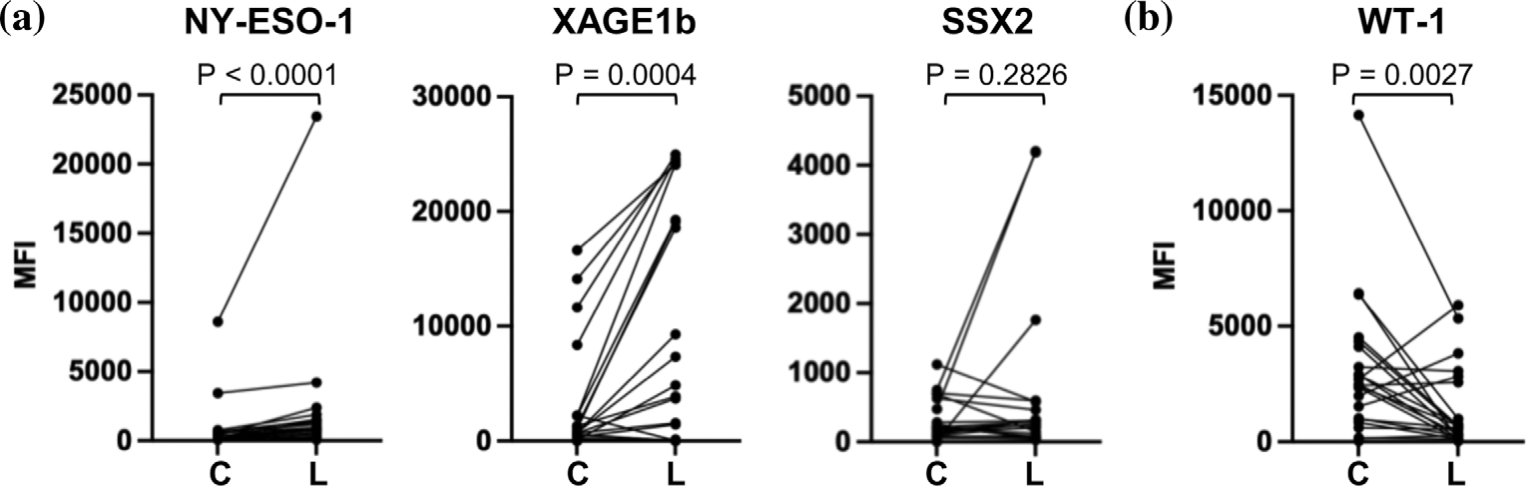
Linear and conformational epitope propensity analysis of autoantibodies in 23 patients with non‐small cell lung cancer. Statistical significance was assessed using the Wilcoxon rank‐sum test. (a) The analysis identified a group of autoantibodies that preferentially targeted the linear epitopes of NY‐ESO‐1, XAGE1b, and SSX2. Autoantibody titers were quantified using glutathione S‐transferase‐fused native and conformational antigens (*C*) and *S*‐cationized denatured and linearized antigens (*L*) in a simultaneous assay with Luminex beads. Titers from identical serum samples are linked by a line for comparison. (b) Conformational epitope‐preferring autoantibodies against WT‐1 were assessed using the same methodology. MFI, mean fluorescence intensity.

Autoantibody‐binding propensities were systematically compared across four autoantibody specificities that recognized linear epitopes, with titers measured against GST‐fused antigens presenting conformational epitopes. The CTAs, NY‐ESO‐1 and XAGE‐1b, consistently showed higher reactivity toward linear epitopes (*p* < 0.0001 and *p* = 0.0004, respectively), whereas SSX2 showed weak reactivity (*p* = 0.2826) (Figure 2a). This observation is consistent with the well‐established structural features of CTAs, which are characterized by abundant IDRs (Figure S2a) (Rajagopalan et al., 2011; Sakaguchi et al., 2026). *S*‐cationization enhances antigen water solubility and conformational flexibility, possibly by increasing the surface exposure of linear epitopes, thereby potentiating autoantibody binding.

In contrast, autoantibodies targeting WT‐1 demonstrated greater reactivity toward conformational epitopes (Figure 2b). Although the WT‐1 conformation was predicted to contain abundant disordered regions (Figure S2a), its partially ordered conformation may facilitate autoantibody binding (*p* = 0.0027). Analysis of the AlphaFold Protein Structure Database entry (AF‐P19544‐F1) indicated that approximately 30%–35% of the WT‐1 protein forms ordered tertiary structures, predominantly within the C‐terminal zinc‐finger domains, whereas the remaining N‐terminal region is intrinsically disordered. Crucially, the zinc‐finger motifs are stabilized by coordinating zinc ions. While overexpression leads to the formation of IBs due to the disordered N‐terminus, the individual zinc‐finger domains can maintain high local thermodynamic stability and autonomous refolding capacity once released in vivo, thereby serving as a robust conformational template for B‐cell receptors.

**FIGURE 3:**
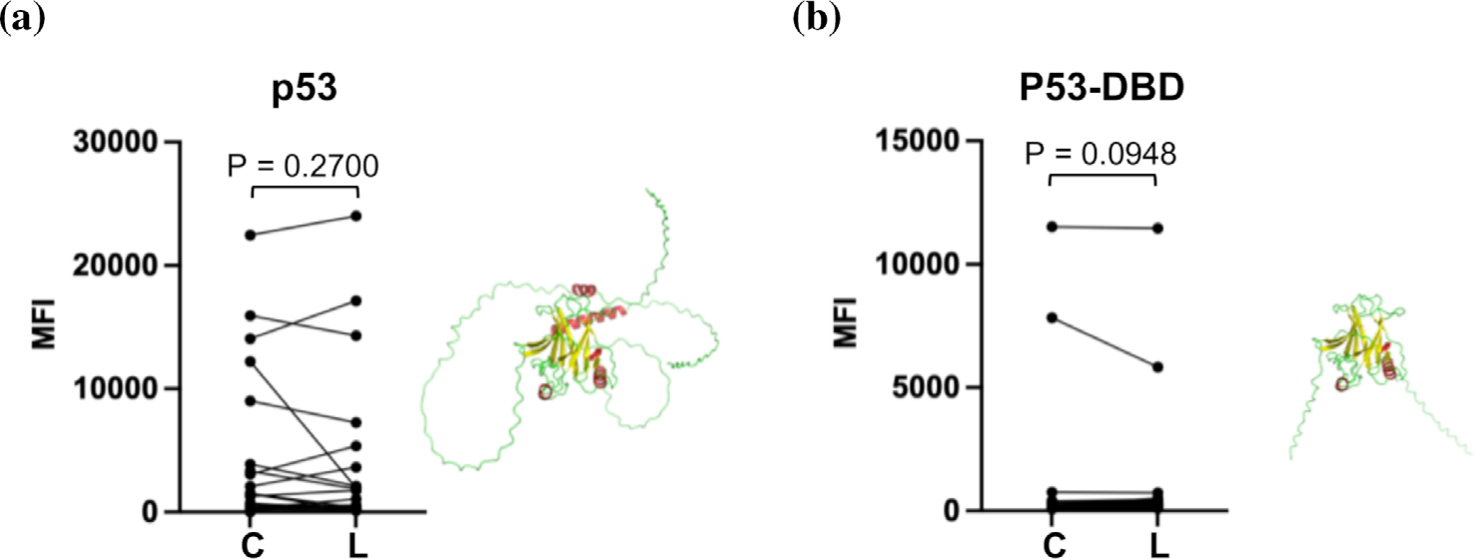
Linear and conformational epitope propensity analysis of anti‐p53 autoantibodies in 23 patients with non‐small cell lung cancer. Statistical significance was assessed using the Wilcoxon rank‐sum test. (a) Autoantibody binding was assessed using antigen‐immobilized Luminex beads coated with full‐length p53 protein, soluble GST‐p53 (*C*), or water‐solubilized and purified *S*‐cationized p53 (*L*). (b) Anti‐p53 autoantibody binding to soluble p53‐DBD (*C*) was compared with that to *S*‐cationized denatured p53‐DBD (*L*). MFI, mean fluorescence intensity.

The epitope characteristics of p53, a tumor suppressor frequently targeted by autoantibodies in NSCLC, were also examined. Autoantibody reactivity toward full‐length p53 was comparable between the denatured *S*‐cationized form (TAP‐p53) and the conformationally intact form (GST‐p53) (Figure 3a). As all 10 cysteine residues are clustered within the p53 DBD, *S*‐cationization is expected to substantially disrupt the native DBD conformation (Figure S2a). The observed equivalence in binding affinities suggests that most p53‐specific autoantibodies in NSCLC recognize linear, rather than conformational, epitopes.

Consistent with this interpretation, autoantibodies directed against isolated p53‐DBD exhibited comparable reactivity toward denatured *S*‐cationized DBD (TAP‐p53‐DBD) (Figure 3b). Structural analysis of p53‐DBD revealed the presence of intrinsically disordered segments at both the N‐ and C‐termini, which likely serve as the primary targets for these autoantibodies. Taken together, these findings support the conclusion that p53 autoantibodies in NSCLC predominantly recognize linear epitopes.

### 2.3 Autoantibody epitope analysis by disrupting conformational epitopes

**FIGURE 4:**
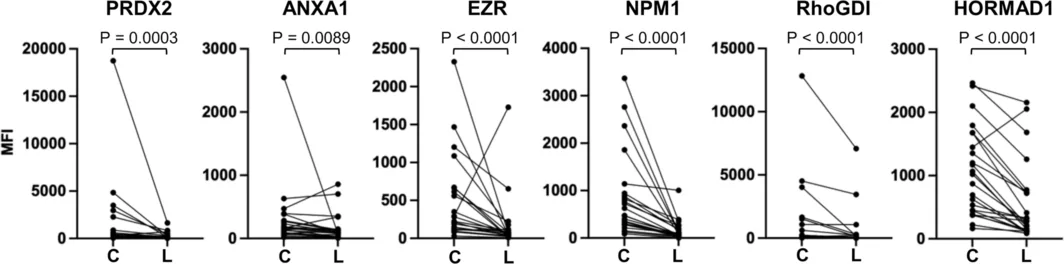
Linear and conformational epitope propensity analyses of stable antigens. The binding properties of autoantibodies were investigated using both native and denatured stable autoantigens. Purified native antigen protein with conformation (*C*) and *S*‐cationized denatured protein exposing linear epitopes (*L*) were immobilized on the color‐coded Luminex beads. Autoantibody titers were simultaneously assessed in diluted non‐small cell lung cancer serum samples (*n* = 23). Statistical significance was assessed using the Wilcoxon rank‐sum test. MFI, mean fluorescence intensity.

To further characterize the propensity of the epitope for autoantibody binding, autoantigens that maintained stable native conformations after expressing in bacteria were subjected to conformational disruption via *S*‐cationization. Autoantibody reactivity toward native conformational antigens presenting conformational epitopes was directly compared with reactivity toward the corresponding *S*‐cationized, denatured, and linearized forms using a Luminex bead‐based assay (Figures 4 and S2b). All six antigens showed a clear preference for conformational epitopes, as evidenced by reduced autoantibody binding after *S*‐cationization‐mediated denaturation (*p* < 0.0001–0.0089). However, most autoantibody reactivity was retained in both native and denatured forms, indicating that the corresponding autoantibodies recognize linear epitopes that are accessible in both conformational states.

Collectively, these results demonstrate that for soluble recombinant autoantigens readily produced in bacterial expression systems, autoantibodies recognize either conformational epitopes on the native protein or linear epitopes present in both native and denatured proteins.

### 2.4 Correlation between structural stability and epitope propensity of autoantigens

Autoantibodies are generated via humoral immune responses against autoantigens released from apoptotic or necrotic cells (Tai & Zheng, 2026). Therefore, the structural properties of these antigens are expected to determine the epitope selectivity of the resulting antibody. We hypothesized that the solubility of recombinant proteins expressed in *E. coli* acts as a reliable proxy for intrinsic structural stability in vivo, provided that the target is a monomeric cytosolic protein that folds autonomously independent of disulfide bond formation, membrane integration, and post‐translational modifications. To test this hypothesis, the soluble‐to‐insoluble fraction ratio obtained from the bacterial expression experiment was used as an empirical measure of thermodynamic stability (Figure S3). The relationship between this experimental solubility and the linear/conformational epitope‐binding ratio (*L*/*C* ratio) is shown in Figure S4.

To provide independent computational validation, the total Rosetta energy unit (REU) scores were calculated from the AlphaFold3‐predicted structures (Abramson et al., 2024) and normalized using the solvent‐accessible surface area (SASA) (Conway et al., 2014; Lyskov et al., 2013; Nivón et al., 2013). To account for variations in protein size when comparing structural stability across different proteins, REU values were divided by SASA, as absolute REU inherently scales with the number of residues. Compared to simple normalization by residue count (REU/residue), dividing by SASA provides a density‐like metric that better accounts for structural compactness and solvent exposure. Although REU/SASA does not constitute an absolute measure of the folding free energy (Δ*G*), it provides a robust and well‐established proxy for the relative thermodynamic stability of the native protein states.

**FIGURE 5:**
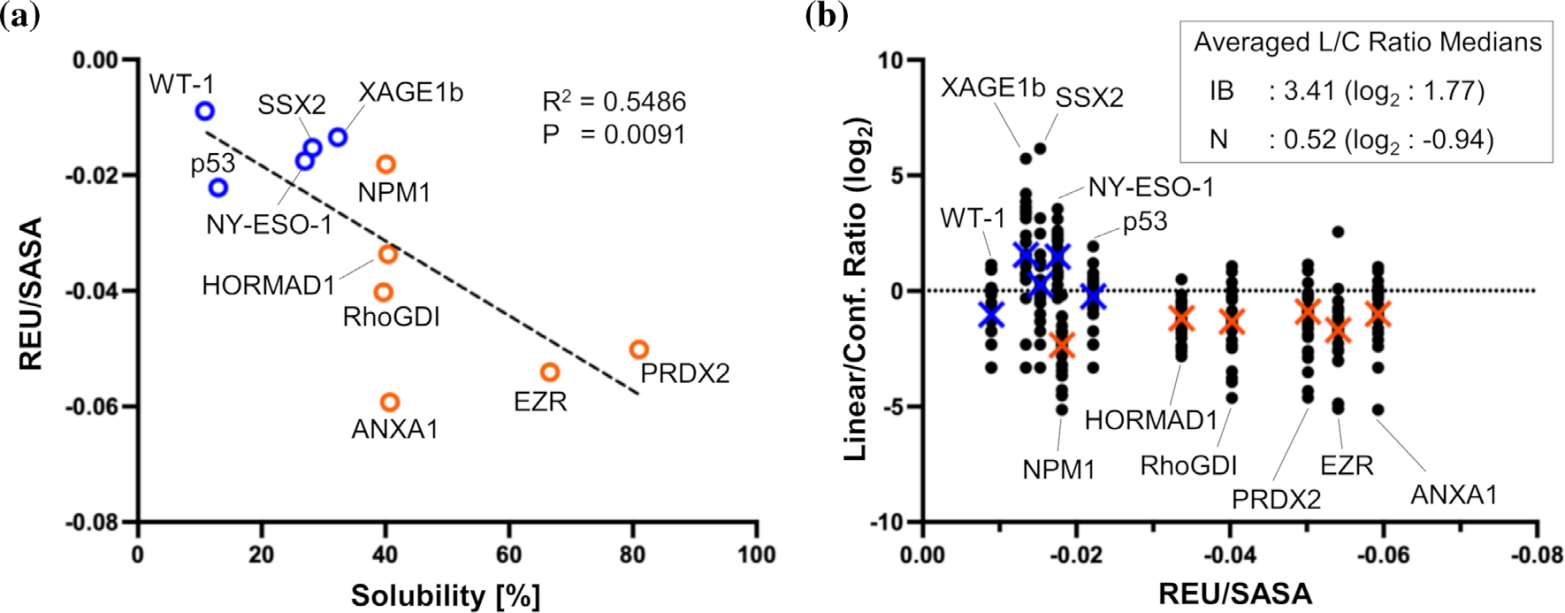
Relationship between epitope propensity of tumor‐associated autoantibodies and conformational stability of target autoantigens. (a) Computational conformational stability (Rosetta Energy Unit/solvent‐accessible surface area [REU/SASA], a proxy for folding Δ*G* derived from predicted structures) was compared with the experimental solubility (%) in *Escherichia coli*. Autoantigens prepared from inclusion body (IB) are shown in blue, while those prepared in the native form from the bacterial cell lysate are shown in orange, on the multiplexed *S*‐cationized antigen‐immobilized Luminex beads assay panel. (b) Computational conformational stability plotted against the propensity of autoantibodies to recognize linear and conformational epitope‐binding ratio (*L*/*C* ratio). The *L*/*C* ratios for the individual sample measurements are represented by filled circles (*n* = 23). The median values are indicated by crosses, with autoantigens prepared from IB shown in blue and those prepared in the native form from the bacterial cell lysate shown in orange. The epitope propensities were quantified as log_2_(*L*/*C*) to symmetrically center equivalent linear and conformational reactivity (*L*/*C* = 1) at zero.

Correlation analysis of the 11 autoantigens revealed a statistically significant relationship between recombinant solubility and REU/SASA (Figure 5a). Autoantigens that were partitioned into IBs and subsequently solubilized by *S*‐cationization exhibited lower REU/SASA values, whereas soluble antigens exhibited higher values. These findings confirmed that the efficiency of expressing human intracellular proteins in a soluble form in bacterial systems accurately reflects their intrinsic conformational stability in vivo.

Plotting of L/C ratio against REU/SASA revealed two discrete clusters (Figure 5b): IB‐forming autoantigens with lower conformational stability showed preferential linear epitope recognition (*L*/*C* ratio: 3.41), whereas soluble autoantigens with higher conformational stability predominantly elicited conformational epitope recognition (*L*/*C* ratio: 0.52). Collectively, these results demonstrate that the autoantibody epitope propensity is tightly coupled to the intrinsic thermodynamic stability of the target autoantigen, which was reliably estimated from both experimental solubility in *E. coli* and computational REU/SASA scoring of the predicted structures.

## 3 DISCUSSION

This study demonstrated that the intrinsic structural stability of the target autoantigen is the primary determinant of epitope propensity in tumor‐associated autoantibodies. As autoantibody production reflects prolonged in vivo immune engagement, thermodynamically stable soluble autoantigens preferentially drive conformational epitope recognition, whereas aggregation‐ or proteolysis‐prone proteins favor linear epitope responses. The concordance between patient‐derived immune data and AlphaFold3‐based REU/SASA estimates confirmed that these epitope propensities are rooted in the fundamental biophysical principles governing protein folding and stability. For monomeric cytosolic autoantigens that fold autonomously in vivo, expression in *E. coli* at 37°C as recombinant proteins provides a tractable experimental proxy for intrinsic conformational stability within the tumor microenvironment. The utility of *E. coli*‐based solubility as a reporter of protein thermodynamic stability is well established across diverse protein engineering and biophysical studies (Dall et al., 2024; Maxwell et al., 1999; Son et al., 2024; Zutz et al., 2021), and our findings extend this framework to the relationship between antigen conformational stability and humoral immune responses in cancer.

This structural instability can be attributed, at least in part, to the ectopic and aberrant expression of CTAs and TAAs. CTAs are normally restricted to immunoprivileged germ cells that integrate into specialized protein complexes (Ahmadi et al., 2021; Cheever et al., 2009; Rajagopalan et al., 2011). However, in cancer cells, genomic dysregulation triggers reactivation in the absence of cognate binding partners. Similarly, TAAs are often overexpressed beyond the stoichiometric balance required for their proper assembly. Consequently, these proteins fail to adopt biologically active conformations, thereby promoting aggregation or proteolytic degradation when cells are lysed by cellular immune reactions, and the proteins leak out to the sites of B‐cell immune reactions. Although most isologous proteins do not elicit immune responses owing to immune tolerance, the breakdown of immune tolerance is often observed with protein‐based biologics owing to aggregation (Rombach‐Riegraf et al., 2014; Swanson et al., 2022). Therefore, aggregation‐prone autoantigens are speculated to preferentially induce humoral immune responses via similar mechanisms.

Our results recapitulate these properties: autoantigens that form IBs in *E. coli*, such as NY‐ESO‐1 and XAGE‐1b, are characterized by high IDR content and possibly lack stable tertiary structures when released from necrotic or apoptotic tumor cells in vivo. In such cases, linear sequences remain the primary structural motifs available for B‐cell recognition, driving the preferential expansion of clones targeting these epitopes in the resulting autoantibody repertoire (Figure 2).

The case with p53 is particularly instructive in this regard. The epitope propensities of autoantibodies targeting the N‐ and C‐terminal segments of p53 have been previously reported (Katchman et al., 2016; Saleh et al., 2004). Although p53 harbors a structured DBD, our data showed that p53‐specific autoantibodies bind equally to both the native and *S*‐cationized denatured forms (Figure 3). As all 10 cysteine residues were concentrated within the DBD, *S*‐cationization selectively disrupted the ordered core while leaving the flanking disordered regions intact. Therefore, the equivalence in binding suggests that the immune response is directed predominantly toward the intrinsically disordered N‐ and C‐terminal segments of p53 despite the presence of a structured domain. This may reflect a broader principle; even in autoantigens containing ordered domains, the humoral immune response preferentially samples disordered solvent‐exposed regions that are constitutively available for B‐cell receptor engagement.

In contrast to p53, WT‐1 exhibits distinct domain‐specific immunogenicity. Although WT‐1 is fractionated into the insoluble pellet in *E. coli* due to its extensive IDRs, the human immune system preferentially recognizes its conformational epitopes (Figure 2b). This apparent discrepancy can be explained by considering the structural autonomies of the constituent domains. The C‐terminal region of WT‐1 contains four tandem C2H2‐type zinc‐finger domains (Wang et al., 2018). These structural motifs may exhibit high local thermodynamic stability upon coordination by zinc ions, thereby enabling them to maintain their structural integrity or refold rapidly, even when full‐length protein aggregates are present in the tumor microenvironment (Fagerlund et al., 2012). While p53 autoantibodies target the IDR extensions, WT‐1 autoantibodies are selectively raised against the structurally robust zinc‐finger cluster. This divergence between p53 and WT‐1 demonstrates that autoantibody epitope propensity is regulated by domain‐specific structural features and local conformational stabilities.

These findings have significant practical implications for designing autoantibody detection platforms. Accurate biomarker measurements require the presentation of antigens in a format that preserves the structural motifs recognized by target antibodies. Assays that rely solely on the native conformations of autoantigens, which are predominantly disordered in vivo, systematically underdetect the dominant antibody repertoire. Conversely, denaturation‐based assays may disrupt primary epitopes of structurally stable autoantigens. Therefore, antigen preparation strategies must be tailored according to the biophysical properties of each target antigen. The MUSCAT assay, which uses chemically stable water‐soluble *S*‐cationized antigens, is particularly well suited for capturing autoantibodies that target disordered CTAs and IDR‐rich TAAs (Futami et al., 2015; Miyamoto et al., 2022; Mori et al., 2025; Sakaguchi et al., 2026).

In summary, the structural integrity of an autoantigen is a primary determinant of its immunological profile. The convergence of observed solubility, in silico REU calculations, and patient‐derived epitope profiling established a robust biophysical framework: autoantigens that are intrinsically disordered owing to ectopic expression or overexpression preferentially elicit linear epitope‐directed antibodies, whereas structurally stable proteins favor conformational responses. These insights clarify the mechanisms governing antitumor humoral immunity and provide a rational evidence‐based foundation for optimizing antigen presentation for the development of sensitive autoantibody‐based cancer diagnostic tools.

## 4 MATERIALS AND METHODS

### 4.1 Preparation and expression of recombinant autoantigens

Recombinant autoantigens (Table S1) were expressed using the pET28b vector (Novagen), which contained an N‐terminal His‐tag (MGSSHHHHHHSSGLVPRGSH) and a C‐terminal Strep‐tag II (GPGWSHPQFEK). For specific constructs, GST‐fused autoantigens were engineered using the pGEX‐4T‐3 vector (Cytiva), which incorporated an N‐terminal GST tag and a C‐terminal Strep‐tag II. These vectors were transformed into *E. coli* BL21(DE3) and cultured at 37°C. Harvested cells were resuspended in lysis buffer (50 mM Tris–HCl, pH 7.5, 50 mM NaCl, and 5 mM MgSO_4_) and disrupted via sonication on ice (three 3‐min cycles; Sonifier 250A, Branson). To reduce lysate viscosity, high‐molecular‐weight bacterial DNA was digested with Benzonase nuclease HC (Millipore). Following centrifugation, the solubility of each autoantigen was determined using densitometric analysis of sodium dodecyl sulfate‐polyacrylamide gels. Soluble autoantigens were purified using immobilized metal affinity chromatography (IMAC) or a StrepTrap XT column (Cytiva) for GST‐fused proteins.

### 4.2 *S*‐cationization and final purification

To prepare denatured antigens with disrupted conformational epitopes, autoantigens were *S*‐cationized. Insoluble autoantigens were solubilized in a buffer containing 6M GdnHCl, 0.1M Tris–HCl (pH 8.0), and 1 mM ethylenediaminetetraacetic acid disodium salt dihydrate. Alternatively, the soluble autoantigens purified using IMAC were adjusted to a final GdnHCl concentration of 6M. For both preparations, cysteines were reduced with 30 mM tris(2‐carboxyethyl) phosphine hydrochloride (Fujifilm‐Wako) for 1.5 h at 37°C, followed by *S‐*alkylation with 60 mM TAP‐Br (Sigma‐Aldrich) for 2 h at 37°C. The reaction was quenched with 0.1 volume of acetic acid and extensively dialyzed in Milli‐Q water. To remove degraded impurities, the *S*‐cationized autoantigens were further purified using reverse‐phase high‐performance liquid chromatography (COSMOSIL Protein‐R, 4.6 mm I.D. × 150 mm, Nacalai Tesque Inc.) using a linear acetonitrile gradient in the presence of 0.1% HCl.

### 4.3 Preparation of autoantigen‐immobilized Luminex beads

Purified autoantigens were immobilized on Bio‐Plex Pro magnetic COOH beads (Bio‐Rad), according to the manufacturer's protocol. To differentiate between epitope types, the same autoantigen was conjugated to beads with distinct spectral addresses (color codes #26, 27, 35, 36, 37, 43, 44, 45, 52, 53, 54, 55, 62, 63, and 64). Native soluble antigens were used as conformational epitope models, whereas *S*‐cationized denatured antigens were used as linear epitope models.

### 4.4 IgG autoantibody binding assay

The autoantibody binding assay was performed in a 96‐well microplate (Greiner BioOne). For each well, 1000 beads per antigen type were pooled to enable the simultaneous evaluation of linear and conformational epitopes. The beads were initially blocked using Block Ace (DS Pharma Biomedical). Following a washing step using a Bio‐Plex Pro wash station (Bio‐Rad), the beads were incubated with plasma samples from patients with NSCLC and diluted 200‐fold in Block Ace. Bound autoantibodies were detected using appropriate secondary antibodies (Miyamoto et al., 2022). For each sample, the reactivity against GST alone was subtracted from that against the corresponding GST‐fusion protein, and the net reactivity to the fused target protein was used for data analysis.

### 4.5 Data acquisition and analysis

Data were acquired using a Bio‐Plex 200 system (Bio‐Rad) and the mean fluorescence intensity (MFI) was determined from a minimum of 50 beads per sample. All MFI values obtained were incorporated for analyzing the overall trend. For a reliable assay, an MFI >100 should be used to ensure a CV <20% (Sakaguchi et al., 2026). However, *L*/*C* ratio‐based comparisons were confirmed to be less affected; therefore, all data were used.

### 4.6 Computational prediction of protein structure and stability

Computational structure prediction and energy‐based scoring were performed to evaluate the intrinsic structural stability of the target autoantigens. Full‐length amino acid sequences, including affinity tags, were used for structural predictions using AlphaFold3 (Abramson et al., 2024). The resultant models, initially in crystallographic information file format, were converted to Protein Data Bank (PDB) format using PyMOL (version 3.1; Schrödinger, LLC) (Figure S2). To refine the predicted structures and minimize energetic clashes, the models were subjected to the Rosetta relaxation protocol (Conway et al., 2014; Lyskov et al., 2013; Nivón et al., 2013) using the Ref2015 forcefield. The total score expressed in REU was extracted from the relaxed structures to indicate the energetic favorability of native folding. To normalize the stability scores across antigens of varying sizes and shapes, SASA was calculated for each relaxed PDB model using the PyMOL software. The calculation was performed using the get_area function with the following parameters: dot_density set to 3, dot_solvent enabled (mode 1), and solvent_radius set to 1.4 Å to represent the water molecules. The final stability index was defined as the total REU normalized to the SASA (REU/SASA ratio). The solubility (%) in *E. coli* was calculated from the band intensities based on Coomassie Brilliant Blue‐stained sodium dodecyl sulfate–polyacrylamide gel electrophoresis  using ImageJ software (version 1.53t). IgG autoantibody reactivity to linear epitopes (*L*) was defined as the reactivity against the *S*‐cationized antigen, whereas reactivity to conformational epitopes (*C*) was defined as the reactivity against the corresponding native antigen maintaining its tertiary structure. The epitope propensities were quantified as log_2_(*L*/*C*) to symmetrically center equivalent linear and conformational reactivity (*L*/*C* = 1) at zero. Positive values indicate preferential recognition of linear epitopes, whereas negative values indicate preferential recognition of conformational epitopes. Correlation analysis was performed using linear regression with GraphPad Prism (version 10.6.1), and the coefficients of determination (*R* ^2^) and corresponding *p*‐values were calculated.

### 4.7 Ethics declarations

Plasma samples from patients with NSCLC were procured from the Okadai Biobank at Okayama University Hospital, Japan, as part of a biomarker screening initiative approved by the Ethics Committee of Okayama University (Application No. 1903‐028), in accordance with the ethical guidelines of the Declaration of Helsinki. Written informed consent for the use of samples and medical records was obtained from all patients who participated in the study.

## AUTHOR CONTRIBUTIONS

**Ai Miyamoto**: Conceptualization, methodology, data curation, formal analysis, investigation, funding acquisition, and writing the original draft. **Rika Yamamoto**: Conceptualization, methodology, data curation, formal analysis, and investigation. **Ryui Sakaguchi**: Conceptualization, methodology, data curation, formal analysis, and investigation. **Rikako Kutsuma**: Methodology and investigation. **Takeru Mori**: data curation and funding acquisition. **Mirei Masui**: Methodology and investigation. **Tomoko Honjo**: Methodology and investigation. **Midori Futami**: Conceptualization. **Mariko Morii**: Conceptualization, methodology, data curation, and funding acquisition. **Hiromi Watanabe**: Data Curation and Resources. **Tadahiro Kuribayashi**: Data Curation and Resources. **Kadoaki Ohashi**: Conceptualization, data curation, and resources. **Katsuyuki Kiura**: Conceptualization, data curation, and resources. **Junichiro Futami**: Conceptualization, methodology, data curation, validation, supervision, formal analysis, funding acquisition, visualization, writing, review, and editing. All authors revised the final manuscript.

## CONFLICT OF INTEREST STATEMENT

The authors declare no conflict of interest.

## Supporting information


**Data S1.** This document includes a list of antigens used in this study (including antigen name, classification as CTA where applicable, UniProt ID, ROSETA Score, SASA, REU/SASA, and solubility), SDS‐PAGE analysis results of the antigens, predicted structural results for each antigen, SDS‐PAGE analysis results used to calculate the protein solubilization efficiency, and the relationship between experimentally determined solubility and the ratio of linear to conformational epitope‐binding ratio (*L*/*C* ratio).

## Data Availability

The datasets generated during the current study are available from the corresponding author on reasonable request.
