# Supplementary material for: Conformational stability and domain‐specific structural features of tumor autoantigens regulate autoantibody epitope propensity
Source: Protein Sci. 2026 Sep 23;35(10):e70808. doi: 10.1002/pro.70808 (PMC13599353; doi:10.1002/pro.70808)
Supplement: Supplementary file 1 [file PRO-35-e70808-s001.pdf]

Supplementary Information to:

Conformational stability and structural modularity of tumor autoantigens dictate autoantibody epitope propensity

Ai Miyamoto[1], Rika Yamamoto[1], Ryui Sakaguchi[1], Rikako Kutsuma[1], Takeru Mori[1], Mirei Masui[1], Tomoko Honjo[1], Midori Futami[2], Mariko Morii[1], Hiromi Watanabe[3], Tadahiro Kuribayashi[3], Kadoaki Ohashi[3], Katsuyuki Kiura[3] and Junichiro Futami[1]*

1 Graduate School of Interdisciplinary Science and Engineering in Health Systems, Okayama University, 3-1-1 Tsushima-Naka, Okayama, 700-8530, Japan.

2 Department of Bioscience, Faculty of Life Science, Okayama University of Science, Okayama, 700-0005, Japan.

3 Department of Respiratory Medicine, Okayama University Hospital, 2-5-1 Shikata-cho, Kita-ku, Okayama 700-8558, Japan

‡ **These two authors contributed equally to this work**

***Corresponding author**

Junichiro Futami, Ph.D.

Graduate School of Interdisciplinary Science and Engineering in Health Systems, Okayama University, 3-1-1 Tsushima-Naka, Okayama, 700-8530, Japan.

Phone & Fax: 81-86-251-8217

E-mail: futamij@okayama-u.ac.jp

Supplementary Information

1. **Table S1** Characteristics of the 11 antigens analyzed in this study
2. **Figure S1** SDS-PAGE analysis of proteins immobilized on MUSCAT assay beads.
3. **Figure S2** Predicted structures of each antigen generated using AlphaFold3.
4. **Figure S3** SDS-PAGE analysis results used for calculation of protein solubilization efficiency.
5. **Figure S4** Relationship between epitope patterns of tumor-associated autoantibodies and the solubility of target autoantigens.

Table S1. Characteristics of the 11 antigens analyzed in this study

This table summarizes the UniProt accession ID, antigen classification, and cancer–testis (CT) antigen number, where applicable, for the 11 antigens analyzed in this study. The number of cysteine residues and the proportion of cysteine relative to the full-length protein sequence were calculated. Structural stability was evaluated using the Rosetta Energy Unit (REU) score, solvent-accessible surface area (SASA), and the normalized REU/SASA ratio calculated from relaxed structures. The experimental solubility (%) in *E.coli* was calculated based on SDS-PAGE band intensities using ImageJ software.

No.	Antigen	Uniprot ID	Classification	CT No	M.W. (kDa)	Cys	Cys (%)	pLDDT<50	pLDDT<50 (%)	ROSETTA Score	SASA	REU/SASA	Solubility (%)
1	NY-ESO-1	P78358-1	CTA	6.1	20.2	5	2.8	114	53.3	-322.0	18345.3	-0.017551775	23.9
2	XAGE1b	Q9HD64-2	CTA	12.1	11.2	4	4.9	26	22.0	-158.7	11816.9	-0.013433081	23.8
3	SSX2	Q16385-1	CTA	5.2	23.8	2	1.1	113	54.3	-320.2	20955.5	-0.015281796	23.1
4	WT-1	P19544-7 (7 - 522 aa)	TAA	-	58.3	18	3.5	391	72.8	-483.5	54222.1	-0.008917853	7.2
5	p53	P04637-1	TAA	-	45.8	10	2.5	152	36.8	-808.3	36458.7	-0.022169389	10.6
6	PRDX2	P32119-1	AA	-	25.3	3	1.3	29	12.7	-668.5	13327.8	-0.050155965	76.8
7	ANXA1	P04083	TAA	-	42.1	4	1.1	41	10.9	-1126.3	18997.3	-0.059288362	37.1
8	EZR	P15311	TAA	-	72.8	2	0.3	35	5.7	-1981.6	36639.2	-0.054082903	74.7
9	NPM1	P06748-1	TAA	-	35.9	3	0.9	127	39.2	-550.8	30380.9	-0.018130413	26.3
10	RhoGDI	P52565-1	TAA	-	26.6	1	0.4	28	12.0	-633.5	15744.5	-0.040237373	42.0
11	HORMAD1	Q86X24-1	CTA	46	47.4	9	2.2	165	39.9	-964.4	28635.5	-0.033677664	34.3

Abbreviations used are as follows: CTA, cancer/testis antigen; TAA, tumor-associated antigen; AA, autoimmune disease-associated antigen.

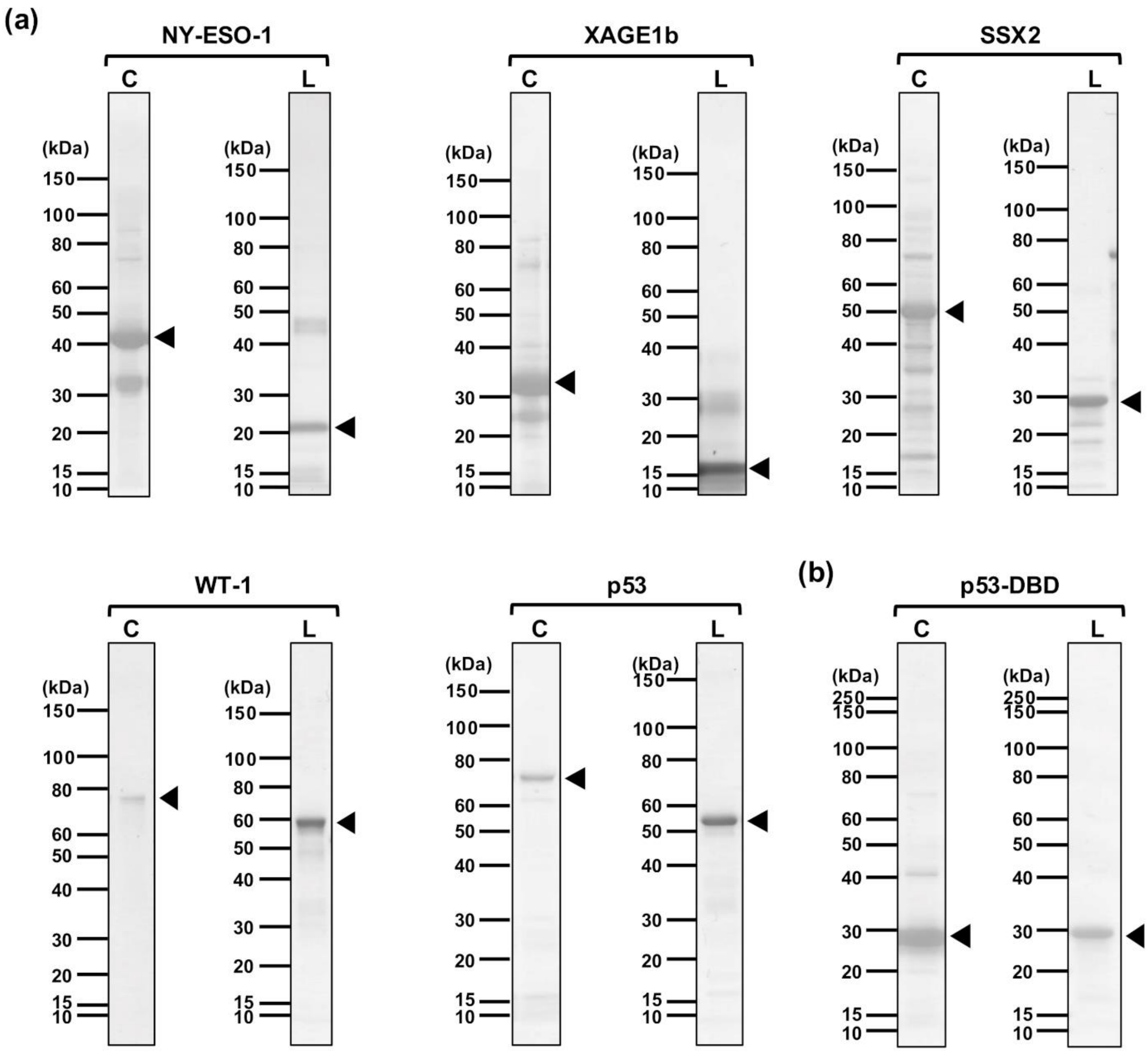
(a)
NY-ESO-1
XAGE1b
SSX2
WT-1
p53
(b)
p53-DBD
C
L
(kDa)
250
150
100
80
60
50
40
30
20
15
10

Figure S1 continued

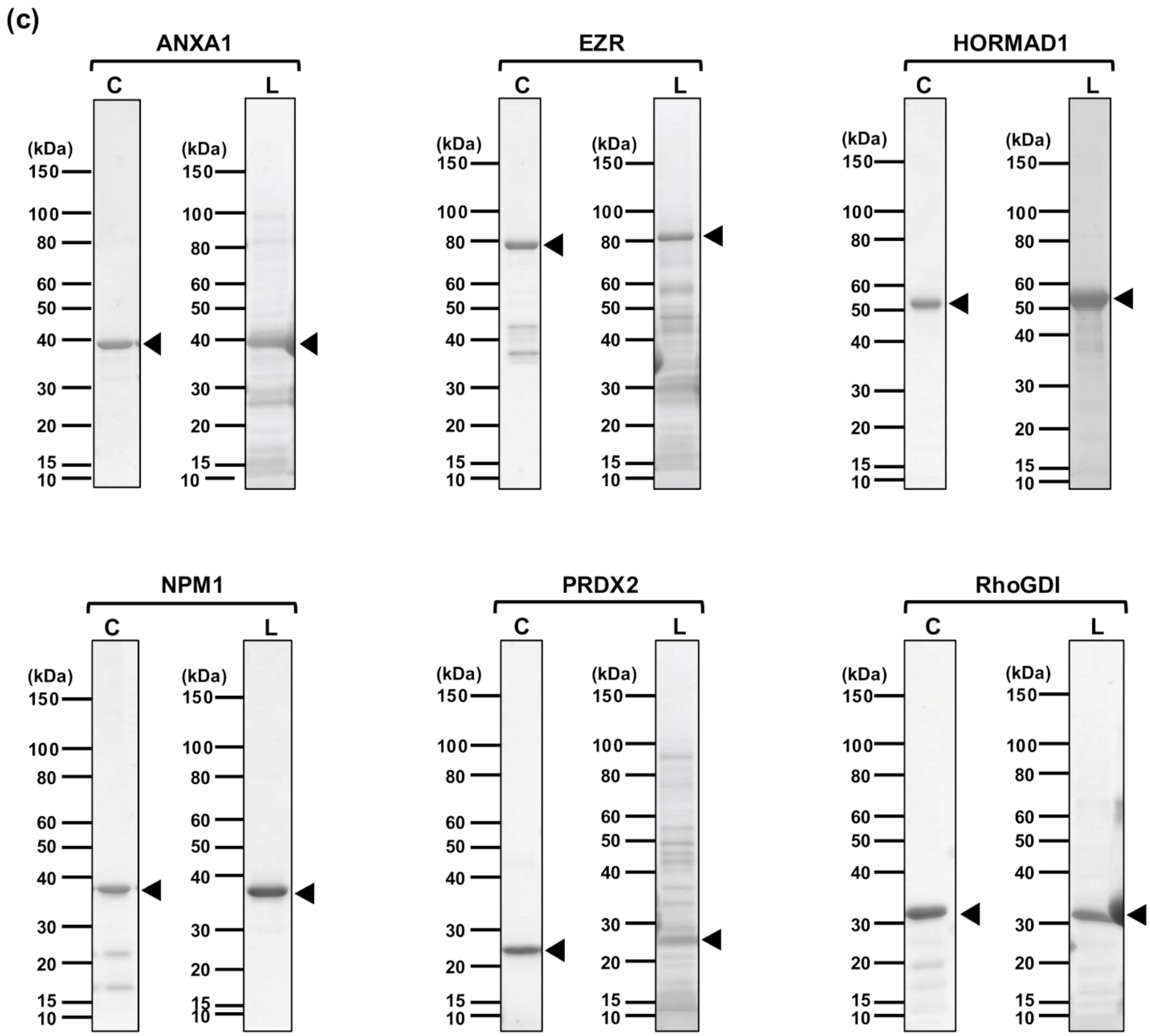

Figure S1. SDS-PAGE analysis of proteins immobilized on MUSCAT assay beads.

(C) indicates native proteins with conformation, whereas (L) indicates *S*-cationized denatured proteins exposing linear epitopes. Proteins were analyzed under reducing conditions and visualized by Coomassie Brilliant Blue (CBB) staining.

(**a**) Recombinant proteins expressed in *E. coli* mainly as inclusion bodies (IBs). Native proteins were obtained by fusing it with GST tag (27.8 kDa) for solubilization.

(**b**) Native (C) and *S*-cationized denatured (L) forms of p53-DBD protein.

(**c**) Recombinant proteins obtainable from the soluble fraction in *E. coli.*

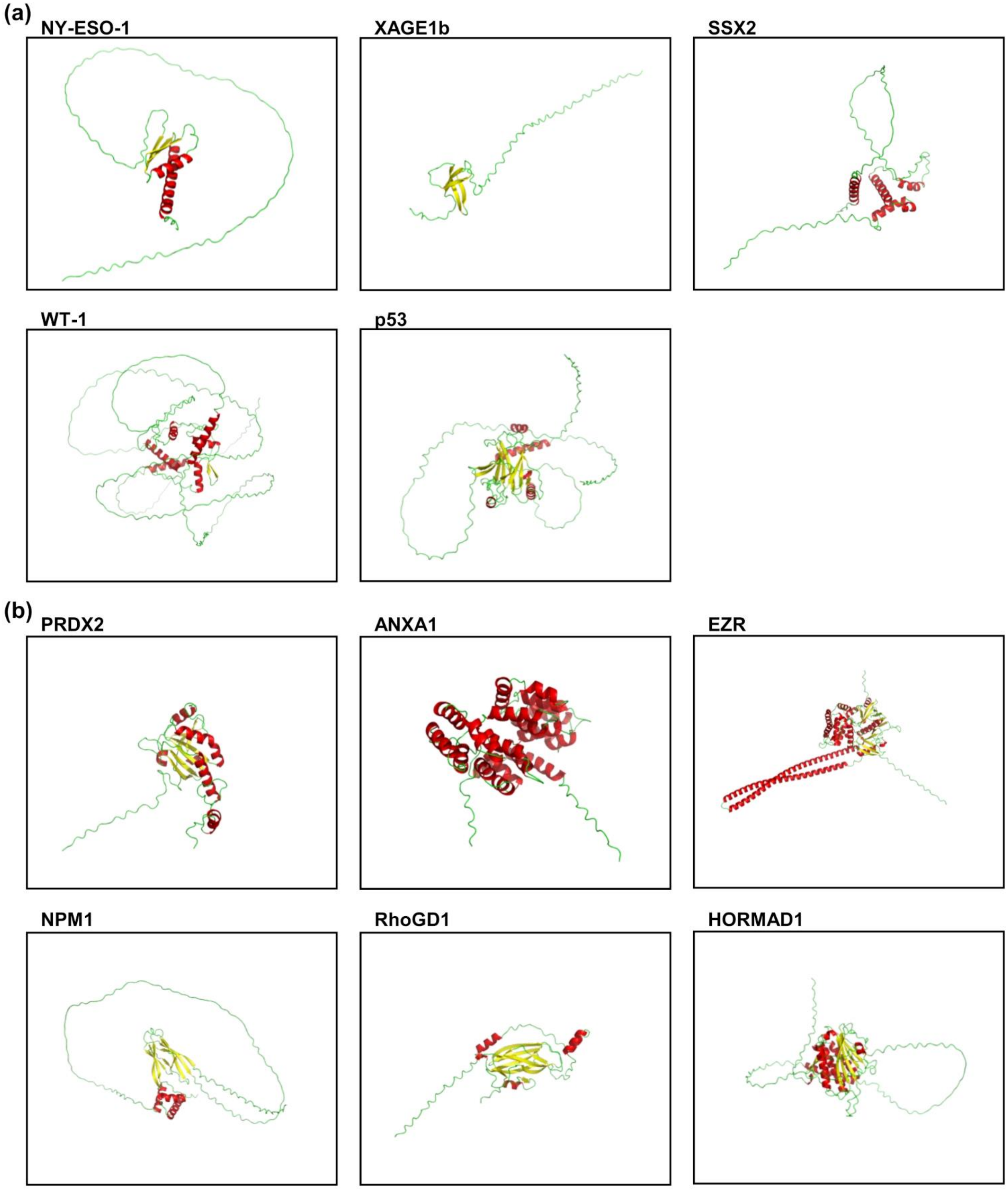

Figure S2. Predicted structures of each antigen generated using AlphaFold3.

In *E. coli* protein expression, proteins that form inclusion bodies are shown in (**a**), and proteins that can be recovered in their native form from the soluble fraction are shown in (**b**).

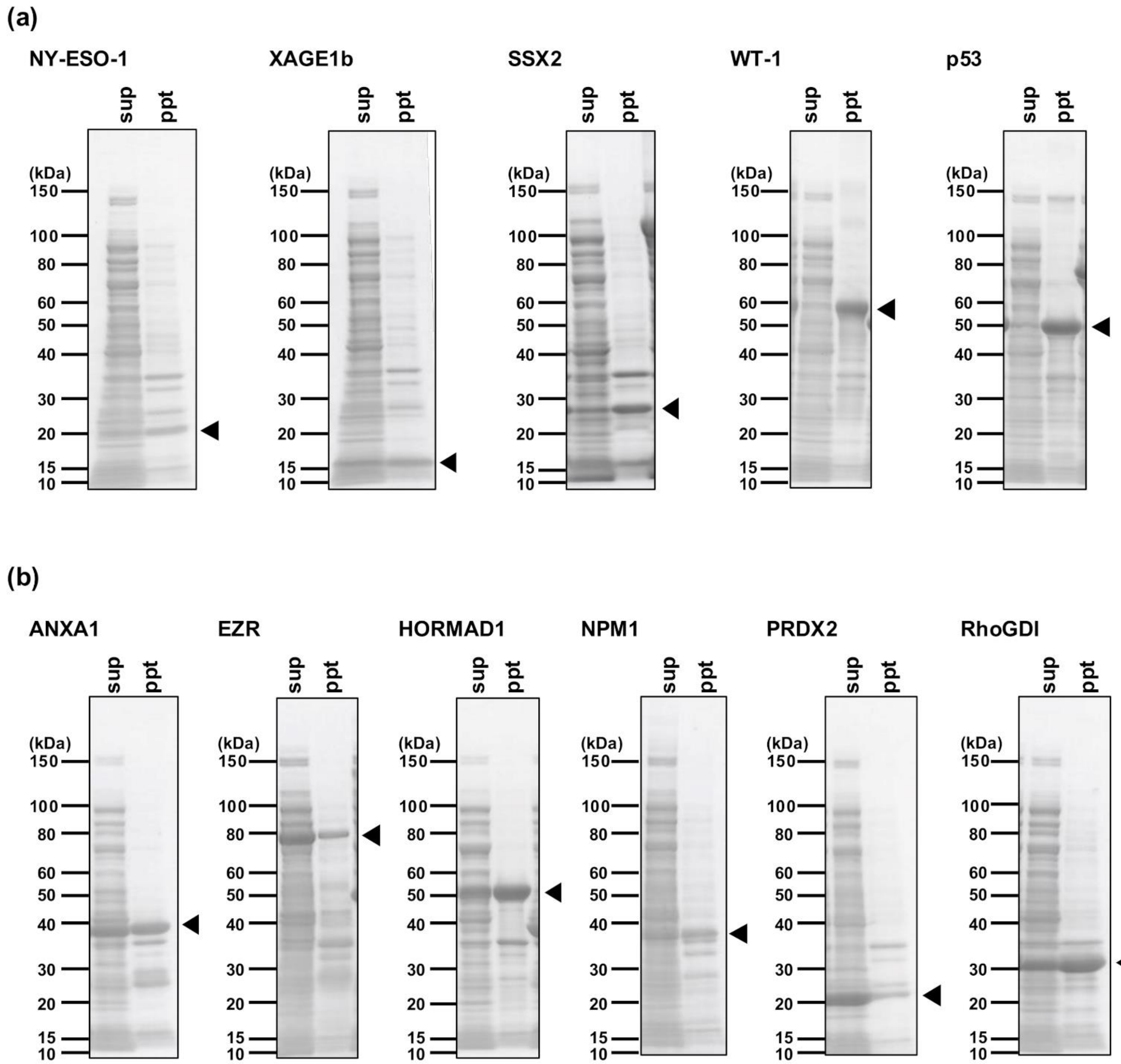

Figure S3. SDS-PAGE analysis results used for calculation of protein solubilization efficiency.

Supernatant and precipitate fractions are indicated as "sup" and "ppt," respectively. In *E. coli* protein expression, proteins that form inclusion bodies are shown in (**a**), and proteins that can be recovered in their native form from the soluble fraction are shown in (**b**). Proteins were analyzed under reducing conditions and visualized by Coomassie Brilliant Blue (CBB) staining.

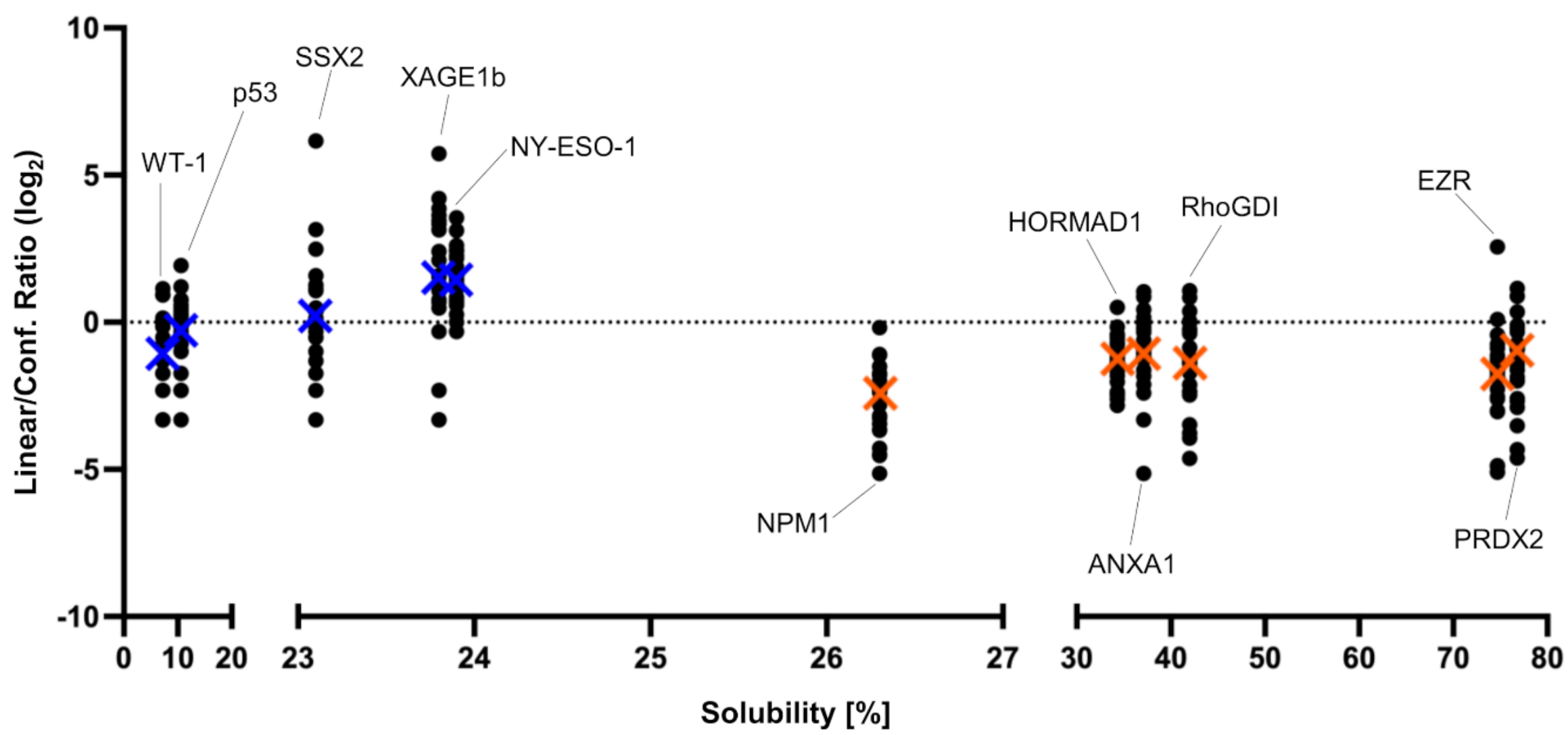

Figure S4. Relationship between epitope patterns of tumor-associated autoantibodies and the solubility of target autoantigens.

Experimental protein solubility in *E. coli* plotted against the propensity of autoantibodies to recognize linear and conformational epitope-binding ratio (L/C ratio). The L/C ratios for the individual sample measurements are represented by filled circles (n=23). The median values are indicated by crosses, with autoantigens prepared from IB shown in blue and those prepared in the native form from the bacterial cell lysate shown in orange.
